# Self-Efficacy, Satisfaction, and Academic Achievement: The Mediator Role of Students' Expectancy-Value Beliefs

**DOI:** 10.3389/fpsyg.2017.01193

**Published:** 2017-07-18

**Authors:** Fernando Doménech-Betoret, Laura Abellán-Roselló, Amparo Gómez-Artiga

**Affiliations:** ^1^Developmental and Educational Psychology, Jaume I University Castellón, Spain; ^2^Developmental and Educational Psychology, University of Valencia Valencia, Spain

**Keywords:** self-efficacy, expectancy-value theory, expectancy beliefs, value beliefs, academic achievement, student satisfaction

## Abstract

Although there is considerable evidence to support the direct effects of self-efficacy beliefs on academic achievement, very few studies have explored the motivational mechanism that mediates the self-efficacy–achievement relationship, and they are necessary to understand how and why self-efficacy affects students' academic achievement. Based on a socio-cognitive perspective of motivation, this study examines the relationships among academic self-efficacy, students' expectancy-value beliefs, teaching process satisfaction, and academic achievement. Its main aim is to identify some motivational-underlying processes through which students' academic self-efficacy affects student achievement and satisfaction. Student achievement and satisfaction are two of the most important learning outcomes, and are considered key indicators of education quality. The sample comprises 797 Spanish secondary education students from 36 educational settings and three schools. The scales that referred to self-efficacy and expectancy-value beliefs were administered at the beginning of the course, while student satisfaction and achievement were measured at the end of the course. The data analysis was conducted by structural equation modeling (SEM). The results revealed that students' expectancy-value beliefs (Subject value, Process expectancy, Achievement expectancy, Cost expectancy) played a mediator role between academic self-efficacy and the achievement/satisfaction relationship. These results provided empirical evidence to better understand the mechanism that mediates self-efficacy–achievement and efficacy–course satisfaction relationships. The implications of these findings for teaching and learning in secondary education are discussed.

## Introduction

Based on a socio-cognitive perspective of motivation, the main purpose of this study is to integrate self-efficacy and expectancy-value beliefs into predicting students' outcomes at secondary schools. This has barely been studied in previous research and sometimes with contradictory results.

Self-efficacy is a key personal variable of Bandura's Social Cognitive Theory (SCT) Bandura's (1986), defined as “an individual's belief in his or her own ability to organize and implement action to produce the desired achievements and results” (Bandura, 1997, p. 3). Educational researchers have paid plenty of attention to this construct (see Michaelides, 2008, for a review). Prior studies have provided strong evidence that self-efficacy is a positive predictor of performance outcomes in different subjects (Schunk et al., 2008; Usher and Pajares, 2008). For instance, Usher and Pajares (2008, p. 751) argued that self-efficacy “predicts students' academic achievement across academic areas and levels.” Despite there being considerable evidence to support the direct effects of self-efficacy beliefs on academic achievement, studies that have explored the motivational mechanism which mediates self-efficacy–achievement relationship are scarce, and are necessary to understand how and why self-efficacy affects students' academic achievement, and will allow instructional actions and programs to improve academic achievement to be designed. One of the most solid proposals that integrate these variables is the social cognitive Expectancy-Value Model (E-VM) of achievement motivation, created by Eccles and her colleagues (Eccles et al., 1983; Wigfield and Eccles, 1992, 2000) based on Atkinson's (1964) expectancy-value model. This complex model includes multiple connections and components that can be classified into three main blocks/categories of variables, arranged in the following sequential order: social world, cognitive processes, and motivational beliefs. All these blocks of variables act directly or indirectly as predictors of students' achievement behavior, persistence, and choice. Centered on motivational beliefs, this model assumes that; first, expectancies for success (achievement expectancy is considered a component of expectancy for success) and subjective task values are directly related to achievement, task choices and persistence; and second, expectancies and task value are assumed to be influenced by individuals' goals and self-schemata. Self-efficacy or personal beliefs of competence is/are considered a salient aspect of self-schemata. Another model that shares similarities with E-VM is the Educational Situation Quality Model (Doménech, 2006, 2011, 2012, 2013; Doménech-Betoret et al., 2014; MOCSE is the acronym in Spanish) because: (a) both models are rooted in the social cognitive perspective of motivation; (b) they emphasize the important role that expectancy-value variables play in predicting students outcomes; (c) self-beliefs constructs (e.g., self-efficacy, self-concept, self-esteem, self-confidence, etc.) are considered important antecedents of expectancy-value variables.

Based on the aforementioned theoretical frameworks, the purpose of this study is to test the validity of a structural model by integrating self-efficacy (adolescent students' self-belief) and expectancy and value constructs into predicting and explaining academic achievement and course satisfaction at secondary school. To examine how these motivational beliefs are related and affect such important students outcomes, they are important to not only design actions and programs to improve teacher effectiveness and students' academic results, but to also contribute to clarify the relationship between self-efficacy and expectancy-value variables in predicting students outcomes, whose results available to date are limited and sometimes contradictory (Williams, 2010).

The term “outcomes” may refer to cognitive and emotional variables. Regarding cognitive variables, learning achievements are considered the most important. As regards emotional variables, satisfaction with a course is an important outcome since it influences students' decisions to continue with or drop out of a course (Levy, 2007). Satisfaction is also an important requirement for successful learning (Sinclaire, 2014). The majority of the considered students' outcomes have to do with cognitive variables such as academic achievement (e.g., grades, test scores, etc.) or learning strategies. In the current study we have decided to include, besides academic achievement, an emotional dependent variable that has been less studied by authors in this tradition, such as, course satisfaction. Academic achievement and course satisfaction are considered two complementary learning outcome as the two face of the same coin. Teachers are interested in knowing not only if their student's progress, but also if they are satisfied with the T–L process followed. Both constructs are important indicators of the quality of the teaching-learning (T–L) process. Therefore, we believe that it would be interesting to test if the selected motivational variables differed in predicting and explaining both academic achievement and students' course satisfaction.

Regarding the relationship between self-efficacy and student satisfaction, Pajares and Schunk (2001) stated that a strong sense of efficacy enhances human well-being; for instance, self-efficacy beliefs influence the amount of stress and anxiety that people experience as they engage in an activity (Pajares and Miller, 1994), and probably when students engage in a course. Self-efficacy also predicts course satisfaction in traditional face-to face classrooms (Bandura, 1997). Although there is empirical evidence to support the positive effects of self-efficacy beliefs on students' well-being and course satisfaction (DeWitz and Walsh, 2002), the motivational mechanisms that mediate the self-efficacy–students satisfaction relationship is still a problem to be solved. Very few studies have centered on examining the mechanism that mediates the self-efficacy–students' course satisfaction relationship, and are necessary to understand how and why self-efficacy affects students' course satisfaction. These findings could provide important clues to promote student satisfaction. Student satisfaction is related to improved academic performance and the decision to take additional classes (Booker and Rebman, 2005). Moreover, satisfaction at school is fundamental for the judgments that students make of their own general well-being (Cummins and Tomyn, 2011).

### Students' expectancy-value beliefs

The Expectancy-value theory is grounded in the social cognitive perspective of motivation. Psychologists in this tradition argue that individuals' choice, persistence, and vigor expended in performance can be predicted and explained basically by expectations of achievement and the value attributed to a task; i.e., by their beliefs about how well they will do in the task and the extent to which they value the task (Atkinson, 1957; Wigfield and Eccles, 1992; Wigfield, 1994). Apart from the components noted above, some theorists from this tradition have introduced a third construct related to the feelings experienced by students when they do a task (Pintrich and De Groot, 1990). We name this third construct “process expectancy.” In the course/subject matter context, we understood process expectancy to be the positive feelings that students expect to experience in their interaction with their teacher during the course (How will I feel studying this subject?). Indeed experience tells us that no-one starts something that is not worthwhile or when expectations of success are very poor because completing the task in such circumstances is considered a waste of time. Finally, nobody starts a task if they do not expect be feel well during the performance process. Hence these beliefs are considered three important indicators of students' motivation (Pintrich and De Groot, 1990), which specify some underlying motivational mechanisms that lead to the initiation and maintenance of action (Pintrich and Schunk, 1996). Centered on a course-subject and based on the above arguments, we herein used three types of beliefs that can make adolescent students decide on striving to learn a subject or not: (a) the subject value (What value does this subject have for me?), (b) the achievement expectancy (Will I be able to pass this subject?), and (c) process expectancy (How will I feel studying this subject?).

The modern Expectancy-value theory (Eccles and Wigfield, 2002; Eccles, 2009) distinguishes four task-value components: attainment value, intrinsic value, utility value and cost. For the present study, which centered on a course subject, we used extrinsic value (which encompasses utility, importance, and interestingness) and cost-benefit components to assess the subject matter value. Eccles and Wigfield (2002) identified cost as a critical component of value, which was conceptualized as a negative determinant in engaging a task due, for instance, to performance anxiety and fear of failure, and to the amount of effort needed to succeed (Eccles and Wigfield, 2002). However, when we centered on a course, we understood that being involved in a specific subject depends not only on the time and effort invested, but also on the benefits (e.g., in terms of results, reinforcements, enjoyment, etc.) that students can obtain. In short, the subject value items employed in this work refer to: (a) the extrinsic subject value, i.e., the perceived utility, importance, and interestingness of the subject (What value does this subject have for me?); (b) the expected cost-benefit relationship to pass the subject (Will it be worth the time and effort that I will have to invest to pass the subject?).

Students' expectancy-value beliefs may have been generated before classes began, from previous experiences, or may arise on the first days of class when students meet the teacher and find out about the study syllabus, evaluation requirements, teacher methodology, etc. (Doménech, 2006, 2011, 2012, 2013). This means that these beliefs can be evaluated at the beginning of the course after some days/week of class.

### The mediator role of expectancy-value beliefs between self-efficacy and the achievement/satisfaction relationship

#### Regarding the relationship between self-efficacy and expectancy-value beliefs

When students face a new academic task, they ask themselves “Can I perform this task?” (self-efficacy) and “Why should I do this task?” (task value). If their answer to the first question is “yes,” they proceed to the next question (Keskin, 2014). This reasoning suggests that self-efficacy is considered a predictor of task value, and not vice versa. Previous studies have demonstrated not only a positive relationship between both constructs (Bong, 2001; Seo and Taherbhai, 2009), but also that self-efficacy is a direct predictor of task value (Kozanitis et al., 2007; Azar et al., 2010; Keskin, 2014).

Prior research has also revealed significant and substantial direct effects of students' self-efficacy on academic expectations (Chemers et al., 2001; Lent et al., 2008). According to these authors, students with high self-efficacy have greater academic expectations and display better academic performance that with low self-efficacy. These findings are consistent with what Bandura's postulated Bandura's (1997) when he argued that self-efficacy is causally prior to outcome expectancy as the results that individuals anticipate depend mainly on their judgments of how well they would be able to perform in a given situation (Bandura, 1997). Therefore, it is assumed that self-efficacy (defined as the perceived capability to perform a given behavior) causally influences expected outcomes of behavior, but not vice versa.

In short, as regards the relationship between self-efficacy and the expectancy-value variables, the above-described findings support the notion that competence beliefs may drive students' expectations and task/subject values in the school context. However, more studies are needed to understand the connections between students' self-beliefs (e.g., self-efficacy, self-concept, self-esteem, etc.) and expectancy-value variables.

#### Regarding the relationship between expectancy-value beliefs and achievement

Prior research has provided empirical evidence which indicates that expectancies and task-values are related to academic choices and achievement in specific domains, such as mathematics (Marsh and Yeung, 1997; Spinath et al., 2004) and language arts (Spinath et al., 2004). Recent cross-sectional and longitudinal studies have found that expectancy beliefs strongly influence achievement, whereas subject value considerably impacts choice, effort and persistence (Nagengast et al., 2011; Gasco and Villarroel, 2014; Guo et al., 2015).

#### Regarding the relationship between expectancy-value beliefs and satisfaction

Less is known about how students' expectancy-value beliefs relate to emotional outcomes, such as student satisfaction. Despite the findings being limited, they seem to support that satisfaction is well explained by task value (Artino, 2008; Diep et al., 2016) and by grade expectancies (Svanum and Aigner, 2011). Nonetheless, most authors highlight the teacher role and teacher–student interaction (Wu et al., 2010) in relation to instructional and emotional supports as the main responsible factors of students' course satisfaction. Accordingly, process expectations, specifically related to the feelings that student experience during their interaction with the teacher, may play the most salient role to explain students' satisfaction. However, the process expectation formed by students can be influenced, in turn, by self-efficacy beliefs. Students with strong self-efficacy beliefs visualize success scenarios, which provide supportive resources, and guidance for performance (Bandura, 1993). As a result, these students tend to experience more satisfaction with the teaching process than the students with low self-efficacy.

Finally, taken all de variables simultaneously, structural models tested in previous studies, based on the expectancy-value theory, provide additional and important evidence to support the mediator role played by motivational expectancy-value variables in the relationship between students' self-beliefs (e.g., self-efficacy, self-concept, self-esteem, etc.) and students outcomes. For example, the study conducted by Doménech-Betoret et al. (2014) in the university context revealed that students' academic self-efficacy had a significant and direct effect on achievement expectations, enjoyable learning expectations and expected dedication (cost) and, in turn, achievement expectation had a significant and direct effect on avoidance strategies (students' outcomes). In addition, subject value had a significant and direct effect on avoidance strategies (students' outcomes). In a similar vein, the study conducted by Bong et al. (2012) in the school context found that the task value and test anxiety significantly mediated the relationships of self-efficacy to achievement.

Based on the aforementioned empirical evidence, it is plausible to assume that the motivators beliefs which derive from the Expectancy-value theory may mediate the relationship between self-efficacy and learning outcomes; e.g., academic achievement and student satisfaction.

### Objectives and hypotheses

**Figure 1:**
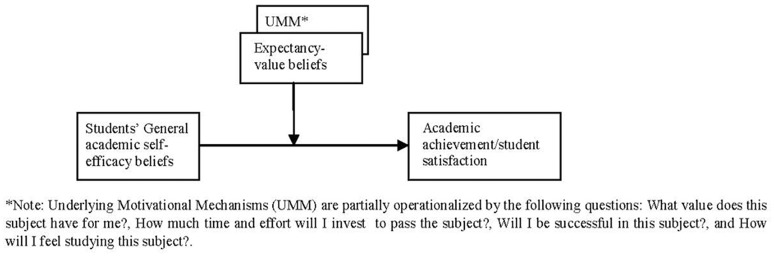
Grafical representation of the study. ^*^Underlying Motivational Mechanisms (UMM) are partially operationalized by the following questions: What value does this subject have for me?, How much time and effort will I invest to pass the subject?, Will I be successful in this subject?, and How will I feel studying this subject?.

According to the aforementioned rationale, the main aim of this study was to examine if the motivational beliefs derived from the Expectancy-value theory play a mediator role between academic self-efficacy and learning outcomes (achievement and satisfaction). At the same time, another aim was to identify some of the motivational processes through which students' academic self-efficacy affects student achievement and satisfaction (see Figure 1). Accordingly, we hypothesized that expectancy-value beliefs would have a direct effect on academic achievement and satisfaction, whereas academic self-efficacy would have an indirect effect on academic achievement and satisfaction through expectancy-value variables. In other words, we predicted that expectancy-value variables would play a mediator role between self-efficacy and achievement (H1), and between self-efficacy and satisfaction (H2). The hypothesized connections were addressed and tested by the Structural Equation Modeling (SEM) procedure with the EQS program (Bentler, 2006). Self-efficacy and expectancy-value variables were all measured in the first academic term after some weeks of class, and student achievement and satisfaction were all measured in the third and last terms of the course. This study can provide new data to improve the motivational theories that integrate self-efficacy, expectancy, and value constructs in the educational setting context, focused on a subject matter as a unit of analysis. It may also help identify the motivational connections that mediate between academic self-efficacy and students' achievement/satisfaction. Important implications for educational practice may derive from these findings since they can provide valuable information to design instructional actions and programs that can improve student achievement and satisfaction. Student achievement and satisfaction are two of the most important learning outcomes, and are also considered key indicators of education quality.

## Materials and methods

### Participants and procedure

The sample consisted of 797 Spanish secondary education students from 36 classes with different subjects, of whom 404 were male (50.7%) and 393 were female (49.3%), and they were aged between 12 and 17 years. Most of their teachers (*N* = 23, 63.88%) also participated in the study, of whom 11 were males (average experience = 29 years) and 12 were females (average experience = 32 years). About 80% of the participating students were Spanish, while the parents of the rest had come from other counties (the majority from Romania, Ecuador, and Morocco) to Spain as emigrants some years ago. One private and two state secondary schools located in east Spain took part in this study, which was carried out at the first four levels of compulsory secondary education: 1st ESO (12–13 years old), 2nd ESO (13–14 years old), 3rd ESO (14–15 years old), and 4th ESO (16–17 years old; ESO is the Spanish acronym for Educación Secundaria Obligatoria—Compulsory Secondary Education). Table 1 displays sample distribution according to levels of education and centers. This study was approved by the Ethics Committee of the Regional Valencian Government, Spain. Consent for students to participate was required from students' parents or legal tutors. Confidentiality and personal data protection were guaranteed in accordance with current Spanish law.

**Table 1:** Characteristics and sample distribution according to courses and centers.

**Year**	**Secondary school**	**Type of center**	**Level**				**Students per center**	**Classrooms per center**
			**1st ESO**	**2nd ESO**	**3rd ESO**	**4th ESO**		
12–13	NSLL	Private	37	35			72	4
12–13	IESLP	Public	37	84	85	32	238	11
13–14	IESFR	Public	169	111	107	100	487	21
Students according to level of education			243	230	192	132	Total = 797	Total = 36

### Measures

The scales used to measure the variables considered herein have been reviewed and refined in previous studies (Doménech, 2006, 2012, 2013; Doménech-Betoret et al., 2014). As most had been designed for university students, they had to be adapted to secondary education in order to use them herein. The scales that referred to self-efficacy and expectancy-value beliefs were administered at time 1 (halfway through the first term), and student satisfaction and achievement were measured at time 2 (halfway through the third term). See Table 2 for item examples.

**Table 2:** Summary of the factor analysis, internal consistency and item example of the scales.

**Scales**	**Factors**	**Items (*n*)**	***M***	***S.D*.**	**Variance**	**Cronbach's α**	**Item examples**
**General academic self-efficacy**	7	25			62.17		
F1: Study techniques		4	2.88	0.81	10.08	0.77	“How good are you at making summaries to help you study?”
F2: Planning and organization		3	2.58	0.83	9.56	0.82	“How well do you plan your work and study?”
F3: Team work skills		4	3.21	0.75	9.41	0.74	“How well do you cope with teamwork with colleagues?”
F4: Coping with new technologies		4	3.18	0.69	8.87	0.73	“How good are you at looking for information on the Internet for your classwork?”
F5: Memorization capacity		3	2.86	0.80	8.55	0.74	“How well do you memorize what you study for an exam?”
F6: Oral and writing communication		4	2.92	0.87	8.36	0.61	“How well do you express what you want to say in writing?”
F7: Coping with exam situations and stress		3	2.67	0.92	7.31	0.70	“How do you cope in exam situations?”
**Expectancy-value beliefs**	4	13			77.06		
F1: Cost expectancy		4	2.37	1.05	19.43	0.90	“Will the time and effort you must invest to pass this subject be too much according to the importance you attach to this subject?”
F2: Achievement expectancy		3	2.90	0.95	14.50	0.85	“Do you think you will be able to obtain good marks for this subject?”
F3: Process expectancy		3	3.24	0.89	14.25	0.83	“Do you think you will feel well treated by the teacher during the course?”
F4: Subject value		3	3.02	0.84	13.52	0.79	“How useful is this subject for you?”
**Satisfaction**							
F1: Students' satisfaction of the Teaching Process		5	3.05	0.92	56.05	0.81	“Are you satisfied with the help and guidelines the teacher provided to complete your classwork and tasks?”
**Student's achievement**							Student' marks ranged from 1 (minimum) to 10 (maximum)

#### Students' general academic self-efficacy scale (25 items, α = 0.86)

This scale is based on the original scales created by Bandura (1990) and by Pastorelli et al. (2001). This scale was used to assess students' self-perception of how competent they were in the academic field. Students indicated their level of agreement within the 1 (very bad) to 4 (very good) range.

#### Expectancy-value scale (13 items, α = 0.78)

This scale comprises 13 items and was designed to measure expectancy-value constructs at the beginning of the teaching-learning process. It was structured and designed according to the Motivational Theory proposed by Pintrich (1989) and Pintrich and De Groot (1990). Students indicated their level of agreement on a 5-point Likert scale within the 1 (I am absolutely unconvinced) to 5 (I am absolutely convinced) range.

#### Satisfaction of the teaching process scale (5 items, α = 0.81)

The original scale was designed by Doménech (2011, 2012) to assess university students' satisfaction with the teaching process followed in the classroom for a specific subject matter. The scale used herein was composed of five items and is a short version of the original teaching process scale adapted to secondary education. Students indicated their level of satisfaction with the teaching process, and opinions were viewed on a 4-point Likert scale within the 1 (unsatisfied) to 5 (very satisfy) range. Finally, student achievement was measured with the marks obtained by students for the first and second academic terms. The mark expected for the third term was also required. Achievement scores ranged from 1 (minimum) to 10 (maximum).

### Statistical analyses

The hypothesized connections were tested by the SEM procedure. The ML and ML robust method of estimation (if the assumption of multivariate normal distribution was violated), developed by Satorra and Bentler (1988, 1994), was used with the EQS program (Bentler, 2006) to calculate the fit indices of the hypothesized models. Since the Chi-square test is sensitive to sample size, using relative fit indices like CFI, the NNFI, and RMSEA is highly recommended (Bentler, 1990). Values below 0.05 for RMSEA indicate a good fit, whereas values up to 0.08 denote an unacceptable fit (Browne and Cudeck, 1993). NNFI- and CFI-values above 0.90 (Hoyle, 1995), or even 0.95 (Hu and Bentler, 1999), were fixed as the cutting-off point.

## Results

### Validity of the measurement model of latent variables

Hypothesized covariance structure models represent only approaches of reality because the obtained indices may be driven by the sample characteristics on which the model was tested (Cudeck and Browne, 1983). One approach to mitigate this limitation is to employ the cross-validation strategy (Byrne, 2012). To apply this strategy, the total sample (*N* = 797) was randomly split into two equivalent subsamples (the calibration sample and the validation sample), following the recommendations of Cudeck and Browne (1983). First, with subsample 1 (*n* = 399), a separate explorative factor analysis (EFA), using the principal component method with varimax rotation, was conducted on all the scales to estimate their factorial structure. Second, by taking the factors extracted in the EFA as the observational variables, a separate confirmatory factor analysis (CFA) was performed with subsample 2 (*n* = 398) to test the goodness of fit and stability of the measurement models of these scales. These two-handed factorial analyses (exploratory and confirmatory) approach provide strong evidence for the reliability of the factors used as latent variables, and improve the validity of the measurement model (Cudeck and Browne, 1983). Finally, the total sample (*N* = 797) was then used to examine the structural model; i.e., the relationships among the latent variables (see Table 2 for details). When data analyses were performed, the initial sample slightly reduced because 23 students did not complete the entire scales. Missing values were not calculated given the large number of participants.

#### Students' general academic self-efficacy scale (25 items)

Exploratory Factor Analysis (Sample 1). Seven factors that corresponded to the seven academic skills included on the scale were extracted, which accounted for 62.17% of total variance. Cronbach's alpha values ranged between 0.82 (maximum) and 0.61 (minimum).

Confirmatory Factor Analysis (Sample 2). The fit indices values obtained using the ML (χ^2^ = 573.459; *p* = 0.000, *d.f*. = 254; χ^2^/*d.f*. = 2.257; NFI = 0.831; NNFI = 0.878; CFI = 0.897; GFI = 0.892; AGFI = 0.861; RMSEA = 0.056) and ML Robust (Satorra-Bentler scaled χ^2^ = 478.366; *p* = 0.000, *d.f*. = 254; χ^2^/*d.f*. = 1.883; NFI = 0.823; NNFI = 0.890; CFI = 0.907; IFI = 0.909; MFI = 0.754; RMSEA = 0.047) estimation methods indicated that the model fitted the data.

#### Expectancy-value scale (13 items)

Exploratory Factor Analysis (Sample 1). Four factors, which corresponded to the four scale constructs, were extracted, and accounted for 77.06% of total variance. Cronbach's alpha values ranged between 0.90 (maximum) and 0.79 (minimum).

Confirmatory Factor Analysis (Sample 2). The fit indices values obtained using the ML (χ^2^ = 136.238; *p* = 0.000, *d.f*. = 94; χ^2^/*d.f*. = 1.449; NFI = 0.963; NNFI = 0.985; CFI = 0.988; GFI = 0.960; AGFI = 0.942; RMSEA = 0.034) and ML Robust (Satorra-Bentler scaled χ^2^ = 117.663; *p* = 0.000, *d.f*. = 94; χ^2^/*d.f*. = 1.251; NFI = 0.963; NNFI = 0.990; CFI = 0.992; IFI = 0.992; MFI = 0.971; RMSEA = 0.025) estimation methods indicated that the model fitted the data.

#### Satisfaction with the teaching process scale (5 items)

Exploratory Factor Analysis (Sample 1). One factor referred to satisfaction with the teaching process (α = 0.81), and was extracted and accounted for 56.05% of total variance. The confirmatory factorial analysis (Sample 2) was not applicable because only one factor was extracted.

### Procedure for testing mediation

The structural equation analysis was carried out with the whole sample to firstly test the mediation role of the expectancy-value beliefs between the self-efficacy–achievement relationship, and secondly the mediation role of the expectancy-value beliefs between the self-efficacy–satisfaction relationship. The procedure followed to test the mediation effect of the expectancy-value beliefs between self-efficacy and achievement, and also between self-efficacy and satisfaction, was conducted in two steps: first by testing the significant direct effects of latent variable self-efficacy on latent variables achievement (M1A model) and satisfaction (M1S model); second by testing the mediated role of the latent variable expectancy-value beliefs on the self-efficacy–achievement relationship (M2A), and also on the efficacy–satisfaction relationship (M2S). In this case we considered the direct and indirect effects between self-efficacy and achievement/satisfaction simultaneously.

### Testing the mediation effect of expectancy-value beliefs between self-efficacy and achievement (H1)

The M1A model was first tested (direct effects) for the mediation role of the expectancy-value beliefs between self-efficacy and achievement. The fit indices values obtained by the ML method (χ^2^ = 194.52; *p* = 0.0000, *d.f*. = 34; NNFI = 0.93; CFI = 0.95; GFI = 0.92; RMSEA = 0.078) and the ML Robust method of estimation (χ^2^ = 173.19; *p* = 0.0000, *d.f*. = 34; NNFI = 0.93; CFI = 0.95; RMSEA = 0.073) indicated that the model satisfactorily fitted the data. According to the data, academic self-efficacy had a significant effect on achievement. So this prerequisite for mediation to exist was met (Baron and Kenny, 1986).

**Figure 2:**
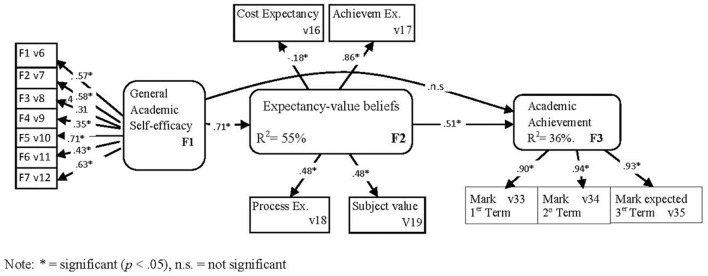
M2A model (direct and indirect effects). Relationship among students' academic self-efficacy, expectancy-value beliefs, and achievement. The structural configuration and standardized coefficients of the model are displayed. ^*^Significant (*p* < 0.05), n.s., not significant.

Next the mediated model M2A was tested. The fit indices values obtained by the ML method (χ^2^ = 329.77; *p* = 0.0000, *d.f*. = 74; NNFI = 0.92; CFI = 0.94; GFI = 0.94; RMSEA = 0.067) and the ML Robust method of estimation (χ^2^ = 293.87; *p* = 0.0000, *d.f*. = 74; NNFI = 0.92; CFI = 0.94; RMSEA = 0.062) indicated that the model fitted the data well. According to the data, latent variable academic self-efficacy had a significant effect on expectancy-value beliefs, which, in turn, had a significant effect on achievement. On the contrary, the path between academic self-efficacy and achievement was not significant. See Figure 2 for details.

### Testing the mediation role of the expectancy-value beliefs between the self-efficacy–satisfaction relationship (H2)

The M1S model was first tested (direct effects) for the mediation role of the expectancy-value beliefs between self-efficacy and satisfaction. The model was optimized when a covariance between two variable errors (E10–E12) from the self-efficacy latent variable was introduced, following the recommendations of the Wald and Lagrange test in the EQS program. Then the model was tested again. The fit indices values obtained by the ML method (χ^2^ = 197.88; *p* = 0.0000, *d.f*. = 52; NNFI = 0.926; CFI = 0.942; GFI = 0.959; RMSEA = 0.060) and the ML Robust method of estimation (χ^2^ = 163.57; *p* = 0.0000, *d.f*. = 52; NNFI = 0.931; CFI = 0.946; RMSEA = 0.053) indicated that the model fitted the data well. According to the data, Academic Self-Efficacy had a significant effect on teaching process satisfaction. So this prerequisite for mediation to exist was met (Baron and Kenny, 1986).

**Figure 3:**
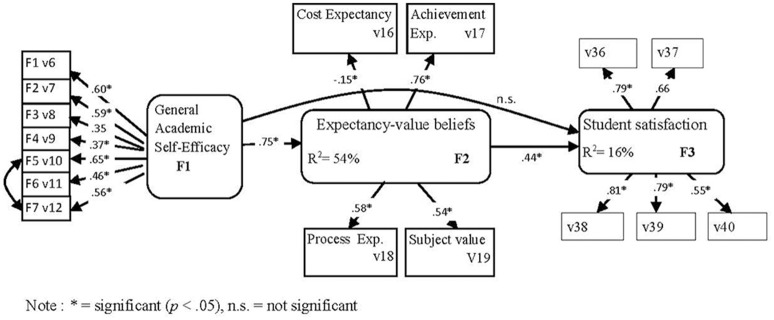
M2S model (direct and indirect effects). Relationship among students' academic self-efficacy, expectancy-value beliefs, and teaching process satisfaction. The structural configuration and standardized coefficients of the optimized model are displayed. ^*^Significant (*p* < 0.05), n.s., not significant.

Second the mediated model M2S was tested. The model was optimized when a covariance between two variable errors (E10–E12) from the self-efficacy latent variable was introduced, following the recommendations of the Wald and Lagrange test in the EQS program. Then the model was tested again. The fit indices values obtained by the ML method (χ^2^ = 399.86; *p* = 0.0000, *d.f*. = 100; NNFI = 0.891; CFI = 0.909; GFI = 0.938; RMSEA = 0.062) and the ML Robust method of estimation (χ^2^ = 343.17; *p* = 0.0000, *d.f*. = 100; NNFI = 0.894; CFI = 0.912; RMSEA = 0.056) indicated that the model fitted the data well. According to the data, the latent variable academic self-efficacy had a significant effect on expectancy-value beliefs which, in turn, had a significant effect on teaching satisfaction. On the contrary, the path between academic self-efficacy and teaching satisfaction was not significant. See Figure 3 for details.

The description and fit indices of the tested models are provided in Table 3, which summarizes the structural equation analyses results.

**Table 3:** Fit indices of the tested models (*N* = 797).

**Model description**	**χ^2^**	***p***	***df***	**NNFI**	**CFI**	**GFI**	**RMSA**
**SELF-EFFICACY AND ACHIEVEMENT**							
**M1A Direct effects**							
ML method	194.52	0.000	34	0.93	0.95	0.92	0.078
ML Robust method	173.19	0.000	34	0.93	0.95		0.073
**M2A Direct and Indirect Effects (Mediation Model)**							
ML method	329.77	0.000	74	0.92	0.94	0.94	0.067
ML Robust method	293.87	0.000	74	0.92	0.94		0.062
**SELF-EFFICACY AND SATISFACTION**							
**M1S Direct Effects**							
ML method	197.88	0.000	52	0.92	0.94	0.96	0.060
ML Robust method	163.57	0.000	52	0.93	0.94		0.053
**M2S Direct And Indirect Effects (Mediation Model)**							
ML method	399.86	0.000	100	0.89	0.91	0.93	0.062
ML Robust Method	343.17	0.000	100	0.89	0.91		0.056

## Discussion

Based on a socio-cognitive perspective of motivation, this study examines; first, the mediator role played by expectancy-value beliefs in the relationship between students' academic self-efficacy and student achievement; second, the mediator role played by expectancy-value beliefs in the relationship between students' academic self-efficacy and student satisfaction with the teaching process. Achievement and satisfaction were considered dependent variables in two separate models as indicators of teaching practice quality.

For the mediator role played by the expectancy-value beliefs in the relationship between students' academic self-efficacy and student achievement, and following the recommendation by Baron and Kenny (1986) for testing mediation, the prediction capacity of students' academic self-efficacy on student achievement was examined first by testing the M1A model; second the mediator role played by expectancy-value beliefs in the relationship between students' academic self-efficacy and student achievement was examined by testing the M2A model. According to the M1A model, the structural analyses indicated a direct, positive and significant effect of the latent variable academic self-efficacy on achievement. According to the M2A model, the obtained fit indices supported the hypothesized connections. This means that the expectancy-value beliefs mediated the relationship between students' academic self-efficacy and student achievement. These results indicated that students' academic self-efficacy affects student achievement, but only indirectly; i.e., by fulfilling the latent variable expectancy-value beliefs.

For the mediator role played by expectancy-value beliefs in the relationship between students' academic self-efficacy and student satisfaction with the teaching process, and following the recommendation by Baron and Kenny (1986) for testing mediation, the prediction capacity of students' academic self-efficacy on student satisfaction was examined first by testing the M1S model; second the mediator role played by expectancy-value beliefs in the relationship between students' academic self-efficacy and student satisfaction was examined by testing the M2S model.

In accordance with the M1S model, the structural analyses indicated a positive and significant direct effect of students' academic self-efficacy on the latent variable teaching process. According to the M2S model, the obtained fit indices supported the hypothesized connections. This means that the expectancy-value beliefs mediated the relationship between students' academic self-efficacy and student satisfaction. These results suggested that students' academic self-efficacy affects student satisfaction, but only indirectly; i.e., by fulfilling the latent variable expectancy-value beliefs.

Given the remarkable variance explained in both models (M2A and M2S), it can be stated that general academic self-efficacy has a strong effect on expectancy-value beliefs. These findings indicated that the level of activation and quality of students' expectancy-value beliefs during the first weeks of the teaching-learning process (after some weeks attending class) depended to a great extent on the evaluation that students made of their own academic skills/capabilities (self-beliefs); e.g., study techniques, planning study, team work skills, coping with new technologies, memorization capacity, oral and written communication, and coping with exam situations. In the light of the obtained results, academic self-efficacy can be considered an important internal source of motivation that is capable of activating students' motivation in the first stage of the behavioral process; i.e., academic self-efficacy contributes to a great extent to activate student students' motivation from the first weeks of the teaching-learning process undertaken with a specific subject. Therefore, it is important to take into account students' academic self-efficacy when they face a new educational setting. These findings are similar to others obtained in previous research. Thus, the study conducted by Doménech-Betoret et al. (2014) revealed the key role played by academic self-efficacy in explaining students' expectations (achievement expectations, enjoyable learning expectations, and expected dedication according to the subject value). In a similar vein, the structural model tested by Bong et al. (2012) revealed that self-beliefs (self-efficacy and self-concept) are good predictors of task value.

Expectancy-value beliefs had a direct positive and significant effect on student achievement/satisfaction. These findings suggested that expectancy-value beliefs (Achievement expectations, Value of the subject matter, Process expectations with the teacher, Expected cost to pass the subject), which were evaluated some weeks after the course began, would be capable of satisfactorily explaining and predicting student achievement and their degree of satisfaction with the teaching process followed with a specific subject matter. The observational variables with higher loadings (Achievement expectations, Value of the subject and Satisfaction expectations with the process) on the latent factor expectancy-value beliefs suggested that these motivational variables were the most important predictors of student achievement and satisfaction. These findings fall in line with previous studies that used the variables from the Expectation-value theory (Guo et al., 2015). The structural model tested by these authors evidenced that Math self-concept (construct used to assess students' expectancy of success) and the Math utility value had a significant and direct effect on students' academic achievement and educational aspirations.

All these findings moved in the expected direction. Academic self-efficacy, considered a general domain variable (Boekaerts, 1999), predicted and explained students' specific expectancy-value beliefs in connection with a specific educational setting. In turn, these specific expectancy-value beliefs predicted/explained students' outcomes (academic achievement and satisfaction). These results were coherent with what Bandura postulated when claiming that specific measures of beliefs were more closely related to behavior (Bandura, 1997).

Based on the obtained results, the following conclusions can be drawn:

- Expectancy-value beliefs, understood as the anticipatory previsions and forecasts that students make in an attempt to anticipate their actions, emotions and results in a new educational situation, were well measured and operationalized by the four motivational selected factors that derived from the Expectancy-value theory: Value of the subject, achievement expectancy, satisfaction expectancy with the process, and the expected cost to pass the subject.
- Students' expectancy-value beliefs, generated/activated during the first weeks of the teaching-learning process, were well explained by the perception or idea that students form about their own basic academic skills. These findings also fall in line with previous studies (Doménech-Betoret et al., 2014).
- Four motivational variables, which mediate the relationship between academic self-efficacy and students' achievement/satisfaction, were identified. These findings fall in line with previous studies, which examined the mediator role of motivational variables in the relationship between self-efficacy and achievement (Bong et al., 2012).
- These findings shed light to better understand the relationship between self-efficacy and expectancy-value beliefs in predicting students' outcomes in secondary education.
- This study allows advances to be made in explaining students' emotional outcomes, such as course satisfaction, which has barely been studied in previous research in the expectancy-value theory context.
- Important educational implications can be derived from the socio-cognitive perspective of motivation acquired from the results obtained to improve students' achievement and satisfaction from a preventive point of view.

### Educational implications

Based on the results obtained in this study, the following educational implications can be made:

First, the expectancy-value beliefs generated during the first weeks of the course are capable of predicting student achievement and their satisfaction with the teaching process followed throughout the course. Therefore, we wish to stress the importance of making a diagnostic evaluation at the beginning of the course of secondary students' expectancy-value beliefs in order to: (a) detect possible shortcomings that students may present in relation to students' expectancy-value beliefs formed at the beginning of the course, after some days of class; (b) design an action plan to overcome or improve these shortcomings.

Second, the obtained results provide evidence that general academic self-efficacy is capable of explaining to a great extent the expectancy-value beliefs formed by secondary students about a specific subject some days after the course starts. Therefore teachers should also take it into account at the very beginning of the course. Accordingly, implementing actions and programs at schools is recommended to improve students' academic skills to, in turn, improve academic self-efficacy. These programs should include a variety of components that fall in line with the sources of self-efficacy beliefs proposed by Bandura (1997) in academic contexts. According to Bandura (1997), self-efficacy beliefs are developed when individuals interpret information from four major sources, such as mastery experience, vicarious experience of observing others, social persuasions that students receive from others, and emotional and psychological states. In the school context, mastery experience refers to the way students interpret and evaluate obtained results, and self-beliefs of competence are revised and created according to these interpretations. Accordingly, teachers should provide instructional scenarios in which students are able to succeed in challenging tasks. Students' judgments of competence are also created by vicarious experience; i.e., by evaluating their capabilities in relation to other students' performance. Another source of self-efficacy is the social persuasions that students receive from others. Accordingly, a supportive message from parents and teachers is important to empower students' self-confidence. Finally, students' self-efficacy is created by their emotional and psychological states as students tend to interpret negative psychological states (stress, anxiety, bad mood, depression, etc.) as evidence for lack of skills, and positive psychological states as indicators of personal competence. Accordingly, promoting students well-being and reducing negative emotional states strengthen students' self-efficacy. For more details about sources of self-efficacy, see the review by Usher and Pajares (2008). In short, the actions and programs that aim to develop students' self-efficacy should be based on these four sources.

We defend the notion of quality education based on a preventive view. Accordingly, we suggest secondary school teachers taking specific actions on the first days of the T–L process to improve adolescent students' beliefs as regards academic self-efficacy (self-beliefs), achievement expectancy, process expectancy and subject value.

- It is important for teachers to strive to transmit the idea that all the students in class are capable of passing the subject matter. This relates with students' psychological need of competence (Deci and Ryan, 1985, 2000).
- From the very beginning of the course, improve students' perception of their own capacity, specifically the general academic skills required to improve progress made at school; e.g., taking actions to bridge some basic gaps in training that some students may still have from former courses, and are necessary to make progress in the subject; or evaluate and recognize the progress made by students since the evaluative feedback that students receive contribute to develop their competence. This point relates with students' psychological need of competence (Deci and Ryan, 1985, 2000).
- Explain to students the value of the subject matter when presenting the subject matter syllabus, and also throughout the course. Inform students about the importance and usefulness of the subject matter (present or future) at the personal, academic and professional levels.
- From the very beginning of the course, promote and take care of the teacher–students interpersonal relationship; e.g., show closeness, respect, and empathy with students throughout the course. This relates with students' psychological need of relatedness (Deci and Ryan, 1985, 2000).

### Limitations and suggestions for future research

Although the results obtained herein are satisfactory, some limitations and suggestions for future research should be pointed out.

First, the results were obtained from schools located in a specific socio-cultural context. Thus, replicating this study in other educational and cultural contexts is recommended to generalize the findings.

Second, extending the tested model should be considered first by introducing other types of self-efficacy, such as metacognitive self-efficacy (Moores et al., 2006) or emotion regulation self-efficacy (Gross and John, 2003); second by including other motivational variables as mediators. Regarding the motivational process, Bandura (1986) distinguished three types of cognitive motivators: (a) causal attributions; (b) outcomes expectations; (c) goals, whose corresponding theories are Attribution theory, Expectancy-value theory, and Goal theory. Accordingly, including new variables as mediators in future research, such as, goal orientation (Pintrich, 2000) or achievement goals, would be interesting (Liem et al., 2008).

Third, although academic self-efficacy and expectancy-value beliefs were measured in the same data collection wave, we provide enough evidence and theoretical support to consider general academic self-efficacy as an antecedent of the students' expectancy-value belief generated in the classroom context.

Fourth, according to Wigfield and Cambria (2010), most of the measures used by researchers to assess motivational beliefs are student self-report measures. However, self-report measures can be problematic, especially for young children or for students who state that school is not important to them. Consequently, we wish to emphasize the importance of combining quantitative and qualitative methods to reduce biases and to obtain more complete information about students' belief.

## Ethics statement

This study was carried out in accordance with the recommendations of the Ethics Committee of the Regional Government of Valencia, Spain, with written informed consent from all subjects. All subjects gave written informed consent in accordance with the Declaration of Helsinki.

## Author contributions

All authors made substantial contributions to design the work, analysis and interpretation of the data, drafting and revising the work, final approval of the version to be published, and finally agreement to be accountable for all aspects of the work in insuring that questions related to the accuracy or integrity of any aspects of the work were appropriately investigated and resolved (FD, LA, and AG).

### Conflict of interest statement

The authors declare that the research was conducted in the absence of any commercial or financial relationships that could be construed as a potential conflict of interest.
